# Ethics education in pediatrics: Implementation and evaluation of an interactive online course for medical students

**DOI:** 10.3205/zma001576

**Published:** 2022-11-15

**Authors:** André Kidszun, Fiona A. Forth, Daniel Matheisl, Franziska Busch, Lara Kaltbeitzel, Sandra Kurz

**Affiliations:** 1 Johannes Gutenberg-University Mainz, University Medical Center, Department of Pediatric and Adolescent Medicine, Division of Neonatology, Germany; 2 University of Bern, Bern University Hospital, Inselspital, Department of Paediatrics, Division of Neonatology, Bern, Switzerland; 3 Johannes Gutenberg-University Mainz, University Medical Center, Institute for the History, Philosophy and Ethics of Medicine, DFG-Research Training Group “Life Sciences – Life Writing”, Mainz, Germany; 4 Medical Center-University of Freiburg, Center for Pediatrics, Department of Neonatology and Pediatric Intensive Care, Freiburg im Breisgau, Germany; 5 Johannes Gutenberg-University Mainz, University Medical Center, Rudolf Frey Lernklinik, Mainz, Germany

**Keywords:** pediatrics, ethics, education, distance-learning, online, interactivity, reflective thinking, medical students

## Abstract

**Introduction:**

The COVID-19 pandemic has catalyzed the development of online learning formats in virtually all areas of medical education. In pediatric ethics, online learning may not only substitute but also offer specific advantages over traditional classroom teaching. Many pediatricians rate their ethics education as poor and medical ethics education lacks evaluation, especially regarding the students’ needs. The aim of this project was to implement and evaluate a novel interactive distance learning approach to engage medical students in pediatric ethics education.

**Methods:**

An online ethics course was designed and delivered between May and June 2020. Core item of this course was a moderated, written forum discussion spanning several days. Evaluation was mixed methods. We evaluated the effectiveness of the course in terms of quality of the learning environment with a particular focus on relevance to students as well as interactive learning and reflective thinking. The Constructivist On-Line Learning Environment Survey (COLLES) was used to evaluate six different domains of the course. Data are presented as mean (standard deviation [SD]). The respective score range is 1-5, whereby a score of 4 or 5 means that the participants indicated the corresponding item as frequently or almost always present.

**Results:**

Responses were available from 104 (78.3%) of the 133 participating students. “Relevance” yielded a score of 4.17 (0.83), “reflective thinking” a score of 4.22 (0.83). “Interactivity” was scored 3.76 (0.99) and “tutor support” 4.72 (0.53). “Peer support” and “interpretation” scored 3.87 (0.98) and 4.49 (0.60), respectively. In qualitative analysis, students particularly valued the structure of the course, the relevance for their professional practice, their active participation and the incentive to reflective thinking. Students also indicated that this was an innovative and exciting format, which fills a current educational gap and should hence be continued beyond the pandemic.

**Conclusion:**

In conclusion, students actively engaged in online learning and perceived this ethics course as highly relevant for their professional practice.

## Zusammenfassung

**Einleitung:**

Die COVID-19-Pandemie hat die Entwicklung von Online-Lernformaten in nahezu allen Bereichen der medizinischen Ausbildung beschleunigt. Im Bereich der pädiatrischen Ethik könnte Onlineunterricht den traditionellen Präsenzunterricht nicht nur ersetzen, sondern diesem gegenüber auch konkrete Vorteile bieten. Viele Pädiaterinnen und Pädiater bezeichnen ihre ethische Ausbildung als schlecht, und der medizinethischen Ausbildung fehlt es an Evaluation, insbesondere hinsichtlich der Bedürfnisse der Studierenden. Das Ziel dieses Projekts war es, einen neuen interaktiven Ansatz für Fernunterricht zu implementieren und evaluieren, mit dem Medizinstudierenden Ethik in der Pädiatrie vermittelt werden soll.

**Methoden:**

Ein Online-Ethikkurs wurde entworfen und von Mai bis Juni 2020 abgehalten. Kernstück dieses Kurses war eine mehrtägige moderierte, schriftliche Forumsdiskussion. Bei der Evaluation wurde ein Methodenmix angewendet. Dabei wurden die Effektivität des Kurses hinsichtlich der Qualität der Lernumgebung unter besonderer Berücksichtigung der Relevanz für die Studierenden sowie des interaktiven Lernens und des reflexiven Denkens evaluiert. Mithilfe des Constructivist On-Line Learning Environment Survey (COLLES; konstruktivistische Bewertung der Online-Lernumgebung) wurden sechs unterschiedliche Aspekte des Kurses bewertet. Die Daten sind als Mittelwert (Standardabweichung [SD]) angegeben. Der jeweilige Wertebereich ist 1-5, wobei die Werte 4 oder 5 anzeigen, dass die Teilnehmenden das jeweilige Item als häufig oder fast immer vorhanden einstuften.

**Ergebnisse:**

Von 104 (78,3%) der insgesamt 133 Studierenden, die an dem Kurs teilnahmen, lagen Antworten vor. „Relevanz“ wurde mit einem Wert von 4,17 (0,83) bewertet, „reflexives Denken“ mit einem Wert von 4,22 (0,83). Der Wert von „Interaktivität“ betrug 3,76 (0,99) und von „Unterstützung durch die Dozentin/den Dozenten“ 4,72 (0,53). „Gegenseitige Unterstützung in der Gruppe“ und „Interpretation“ bewerteten die Teilnehmenden jeweils mit 3,87 (0,98) und 4,49 (0,60). Die qualitative Analyse zeigt, dass die Studierenden vor allem die Struktur des Kurses, die Relevanz für ihre Berufspraxis, ihre aktive Beteiligung sowie den Anreiz zum reflexiven Denken schätzten. Zudem gaben die Studierenden an, dass es sich um ein innovatives und spannendes Format handle, das eine derzeit bestehende Ausbildungslücke schließe und daher auch über die Pandemie hinaus fortgeführt werden solle.

**Schlussfolgerung:**

Abschließend ist festzuhalten, dass sich die Studierenden aktiv am Onlineunterricht beteiligten und sie diesen Ethikkurs als überaus relevant für ihre berufliche Praxis wahrnahmen.

## Introduction

Undergraduate medical ethics education has two main goals: first, to enable future physicians to deal with ethical problems or dilemmas, and second, to provide a prerequisite for good professional conduct as a physician. The need to integrate medical ethics topics into the curriculum of human medicine is widely recognized and increasingly demanded [1]. Worldwide, many countries have incorporated ethics education longitudinally into their medical curricula. However, content, methods, and effectiveness of ethics education in medicine are poorly evaluated [1], [2], [3], [4], [5]. In particular, the needs of medical students and their perceptions of ethics education are still largely unknown. It is reported that face-to-face ethics education takes place and classrooms, often in an unstructured, theoretical form characterized by low learning activity [5], [6]. But given the lack of studies on this topic, no valid assessment can be made, especially for the German-speaking countries. Data are also lacking on training in applied subject-specific ethics, particularly in pediatrics. Studies from the English-speaking world show that many pediatricians rate their ethics education as poor, which raises the question of how training can be improved [7], [8]. In this sense, content and methodology of the medical ethics curriculum require further development and are subject of current research and discussion [1], [6]. The pandemic-induced shift to digital learning formats has triggered an expanded discussion about the best possible learning strategies. Only recently has undergraduate ethics education increasingly been delivered online, with promising initial results [9]. 

The aim of this project was to implement and evaluate a new learner-centered format for applied undergraduate ethics education.

## Course development

### Objective

The aim of this project was to further educate medical students in applied pediatric ethics and to promote and train critical and reflective thinking in the education of future physicians. Due to circumstances of the COVID-19 pandemic an interactive online course was ad hoc developed and evaluated in various aspects. The methodological concept of the course was based on a social constructivist approach, which aims at co-constructing new knowledge, at best, based on students’ own experiences. The students’ own views and attitudes should be presented coherently, but they should also be able to question them critically. The course was intended to combine specific advantages of online learning with those of a practice-oriented medical ethics education. Topics in medical ethics should be dealt with and discussed, based on concrete, subject-specific clinical examples. Both, thematically and conceptually, this course represents a new, previously untested approach in undergraduate ethics education. Regarding its effects, the course was to be evaluated by the participating students using a mixed-methods approach. 

#### Content and structure

An interactive online learning format was set up at the onset of the COVID-19 pandemic in 2020. Distance-learning approaches were rapidly introduced to substitute parts of classroom and bedside teaching to cope with access restrictions to the campus and the university hospital. Participants were fifth-year medical students. Participants hence had prior training in general medical ethics in their 3^rd^ year of study. The course was introduced to replace parts of the pediatric internship, which could not be carried out as planned due to the pandemic. New learning content was defined for this course as pediatric ethics education was not previously part of the curriculum. The course was not graded but attendance was necessary to complete the pediatric internship. Each course unit comprised 16-18 active participants. The duration of the course unit was of one week (Friday-Friday) for the individual participant. 

A case-based teaching and discussion format was created and implemented using an interactive web-based learning platform. Four different course units covering the following ethical topics were designed:

1. distributive justice,
2. the best interest standard,
3. cross-cultural aspects of pediatric care and
4. medical futility.

As the first component of the course, students were asked to read and comment in about 1-2 written pages on a theme-specific pediatric ethics-focused article. To guide the students’ comments, two sets of tasks were provided. The first task was directed to identify and discuss some of the ethical issues presented in the article while the other aimed to encourage students to reflect on and justify their own opinions and to bring in their own experiences. For example, in the case of distributive justice these tasks were:

1. “Please discuss and debate at least one of the problems in pediatric intensive care presented in the article! Which overarching health care problems are addressed? What options for allocating medical resources are identified? Please comment on these!” and
2. “What criteria would you use to triage patients in the event of a shortage of medical resources? Refer to the arguments in the article, but also justify your selection with current examples! In addition to the physician’s perspective, try to illuminate the problem from the patient’s perspective as well!”

Mandatory basic readings were illustrative case-based articles that already contained discussions or different or changing views on ethical issues in pediatrics [10], [11], [12], [13]. Additional scientific articles were provided as optional readings and were supplemented with other resources such as videos, books and newspaper articles. Specific information on each course content and references to potential resources for pediatric ethics education are given in attachment 1 .

Following reading, preparation, and presentation, as a second component of the course, students were nudged to engage in a written online forum discussion by a request of a minimum of two “reasonable” replies to other students’ contributions. Following an introduction on Friday afternoon, students were asked to provide their comments until Wednesday afternoon at the latest. Forum discussion was open for the total duration of the course unit, which again ended on Friday afternoon.

One tutor with professional training in pediatric ethics gave feedback to all students’ remarks and served as a moderator during the discussions. The written feedback was available for all course participants to view, as were all other forum postings. The participants agreed to keep the content of the forum discussion confidential.

#### Methods of evaluation

Evaluation was performed using a mixed-methods approach. We evaluated the effectiveness of the course in terms of quality of the learning environment with a particular focus on relevance to students as well as interactive learning and reflective thinking. Participation was voluntary and anonymous. Quantitative evaluation was carried out after completion of the course, using the “actual form” of the Constructivist On-Line Learning Environment Survey (COLLES) by Taylor and Maor [14] which was included in the survey module of the online learning platform. The COLLES comprises an economical 24 items grouped into six scales. Each scale addresses a key question on the quality of the online learning environment. The scales and the respective questions are as follows:

- Relevance: How relevant is online learning to students' professional practices?
- Reflection: Does online learning stimulate students' critical reflective thinking?
- Interactivity: To what extent do students engage online in rich educative dialogue?
- Tutor support: How well do tutors enable students to participate in online learning?
- Peer support: Is sensitive and encouraging support provided online by fellow students?
- Interpretation: Do students and tutors make good sense of each other's online communications?

The 24 items attributed to the six scales are each rated by use of a fully verbalized 5 point Likert scale with the following scale points: 1=almost never, 2=seldom, 3=sometimes, 4=often, 5=almost always. For each scale, the associated items are subsequently aggregated to a mean score. The mean score for each scale is presented with the respective standard deviation (SD) and score range. The possible score range corresponds to the number of scale points of the Likert scale provided for assessment and is 1-5.

In addition, students were also able to freely comment on the course. The students' free-text answers were subjected to a qualitative content analysis according to Mayring using the software QCAMap, [15]. Categories for the qualitative content analysis were generated inductively. In terms of a quality check, the content was coded twice and double-checked regarding agreement of coding in terms of stability. Subsequently a summative analysis of the developed categories was conducted. A denomination probability was prespecified at 5%. Multiple coding was allowed.

## Results

Over a period of 8 weeks in May and June 2020 the described online course was conducted at the University Medical Center of the Johannes Gutenberg-University Mainz in Mainz, Germany. A total of 133 students completed the week-long course unit. No administrative, technical or organizational problems occurred. 

### Quantitative evaluation

Evaluation was performed by 104 of the 133 (78.3%) students. All scales of the COLLES were analyzed and scores by aggregation of the respective items of each scale. Data are presented as mean (SD). “Relevance” yielded a score of 4.17 (0.83), “reflective thinking” a score of 4.22 (0.83). “Interactivity” was on average scored 3.76 (0.99) and “tutor support” 4.72 (0.53). “Peer support” and “interpretation” were scored 3.87 (0.98) and 4.49 (0.60) on average, respectively. 

Attachment 2 summarizes and illustrates the results separate by the different domains of the COLLES. For each the 6 scales of the COLLES the results of the respective 4 items are shown. 

#### Qualitative evaluation

Qualitative evaluation was provided by 64/104 (61.5%) participants contributing to the overall evaluation. With a denomination probability prespecified at 5%, content was evaluated if mentioned by at least three participants. 

Content analysis revealed 6 main categories:

1. relevance
2. reflective thinking
3. active participation
4. role of ethics online education in the medical curriculum
5. tutor feedback
6. critics and suggestions for improvement

Students particularly valued the structure of the course, the relevance for their professional practice, their active participation and the incentive to reflective thinking. Students also indicated that this was an innovative and exciting format, which fills a current training gap and should therefore be continued beyond the pandemic.

Students’ perceptions of these main categories and respective subcategories are summarized in attachment 3 . Four categories covered by the COLLES (relevance, reflective thinking, interactivity, tutor support) also emerged during qualitative analysis, while two categories of the COLLES did not (peer support and interpretation).

Writing longer text passages was reported as quite a challenge for some students, something that has not been adequately practiced in the medical curriculum. Formulating own opinions and to take a stand in discussions as well as practicing self-reflection were other challenges reported. 

## Discussion

### Relevance 

Young graduates report gaps and deficiencies in medical ethics education. In a survey of young U.S. pediatricians regarding their past experiences with ethics and professionalism education, this is confirmed [16]. The majority of respondents (169 of 295 (57%)) reported that ethics and professionalism were taught in an ad hoc fashion, without an organized curriculum. Nearly all respondents (97%; 285 of 293) believed that ethics and professionalism training was useful in their daily practice. Nearly half (44%; 130 of 294) disagreed with the statement that ethics and professionalism could best be learned by observing experienced physicians. The majority described themselves as competent to address the ethical and professional issues faced in practice, but nonetheless graduates reported gaps in their education. Corresponding studies for the German-speaking countries and many other parts in the world are lacking. It hence remains unclear whether these results are generalizable. 

In terms of relevance for practice, the course presented in this report appeared to meet the needs of the students. Relevance for practice was, however, not only content related but also to the skills that were trained in this course, especially finding, and formulating one’s own position, training to debate and make decisions and to consolidate moral views. Interestingly, it seemed, as if writing a somewhat longer text passage considering the arguments of others as well as own considerations was quite a challenge for some students, something new in the current medical curriculum und hence something that might need to be practiced more intensely. Another important point was the practice of self-reflection that was a rather new experience for some of the participants.

#### Reflective thinking and interactivity

In ethics classes for medical students, activation of participants often seems problematic [17]. While case-based learning is a frequently used form of teaching ethics [18], [19], the effectiveness of this form of teaching can probably be increased by subsequent group discussions [19]. Students definitively favor interaction. Ethics instruction, they say, should be structured but interactive in all cases [20]. Students in this course especially valued interactivity and critical, reflective thinking. Many students indicated that exchange of ideas not only stimulated additional reflective thinking but also motivated for additional personal research, which then in turn was discussed again. This way, knowledge co-construction occurred as a logical consequence. One subtheme of interactivity was ‘enthusiasm and fun’. Some students did not only participate, but also appeared to appreciate this learning approach. Key precondition remains an open, diverse, shame-free and confidential atmosphere.

#### Methodology of the course and ethics education in the medical curriculum 

Online ethics learning may offer new avenues for collaborative, constructive, and critically reflective learning. Online ethics instruction, for example, has already been used successfully on international level, in postgraduate continuing education. Such an approach seems particularly suited to actively engage participants [21]. The increased use of online teaching in medical education requires a deeper investigation of learning methods and suitable learning theories. Social constructivism is one such theory of knowledge transformation [22] and describes an epistemology or way of knowing in which learners reflectively collaborate to co-construct new understandings, particularly in the context of questioning each other based on their personal experience [23]. Initial experience in applying this theory on research ethics for engineers in an online class is available and promising in terms of active learning of specific skills [24].

Here we provide first evidence that the methodological transfer to undergraduate ethics teaching is feasible and desirable. Many students commented that the structure of the course worked extremely well. Some were hesitant in advance, but many convinced in the end. In this course, training in applied ethics added to und built up on already acquired basic ethical knowledge and skills. The course appeared to be well designed for senior medical students. Nevertheless, a fixed implementation of such a course requires careful alignment with existing local educational structures, learning concepts, exams and other educational opportunities. 

An important result of this evaluation was the finding that this distance-learning approach appears to have specific advantages over traditional classroom teaching. The week-long discussion forum offered more room to deeply delve into problems, gave time to ponder, time to formulate answers and time to understand and respond. Several participants suggested to continue offering the course to subsequent students as the issues appearing in the course were not well covered in the current local curriculum. 

#### Tutor support

Tutor support appears to be of critical importance in modeling the discussion and giving support. The latter requires specialized education, which may be not readily available at many centers. Standardized tutor support and tutor training would also be necessary. It is to be considered that the training of tutors as well as the tutoring itself, inter alia the giving of feedback on-time, is felt to be extremely time consuming. 

#### Critics and suggestions for improvement

Participants were mainly worried about missing subject-specific pediatrics education, which was an issue during learning in the pandemic. Of course, online ethics education cannot replace other content but may be implemented as a relevant complement. The scope of the course needs to be clearly defined and communicated. Several participants also asked for a final clear take home message, more and more specific themes and examples, at best from the local clinic or hospital, i.e., personal experiences from physicians in practice. Another suggestion was to conclude the course with real-time video feedback. In the further development, it could be fruitful to integrate these learners’ suggestions.

#### Limitations

First, generalizability of our findings is clearly limited. This was a single-center experience performed under the specific circumstances of the COVID-19 pandemic including a limited number of participants and in the absence of a control group. Second, the success of implementation was based solely on student self-assessment, not on objective measures of effectiveness. Further studies using rigorous research designs and including larger numbers of participants at multiple sites are necessary to confirm our findings. While technical transferability is quite easily given, it is hampered by the need to integrate such a course into existing educational programs, which might be substantially different from the one in this report. Tutor performance being the other critical component of transferability requires further investigation, as do the role of peer support and content interpretation.

## Conclusion

Here we report on the successful implementation of an interactive online pediatric ethics course. The implementation of a learning format including the theoretical (knowledge acquisition) and practical (application of ethical principles, critical reflection) consideration of medical ethics topics represents an innovative thematic approach in the context of medical studies. Also new is the approach to teach this topic in an online format. The project presented has the potential to promote critical and reflective thinking, and thus to enrich the education of future physicians by an important aspect. The need to develop, argue, and discuss firm ethical positions in addition to professional expertise is undeniable. In this approach, the distance-learning format is at best not a stopgap, but a new, previously unused opportunity for interaction. This kind of interaction allows for in-depth consideration of others’ arguments and time to develop and, if necessary, substantiate one’s own response. Moreover, the format allows for transfer to other sites or other faculty. 

## Acknowledgements

The first author would like to thank Barbara Kidszun for her generous support during course development and implementation. 

## Competing interests

The authors declare that they have no competing interests. 

## Supplementary Material

Course content and potential additional resources

Summary of the results of the Constructivist On-Line Learning Environment Survey (COLLES)

Summary of the students’ perceptions derived from the 64 answers on the final open evaluative question: “Do you have any other comments?”
