# Supplementary material for: Ethics education in pediatrics: Implementation and evaluation of an interactive online course for medical students
Source: GMS J Med Educ. 2022 Nov 15;39(5):Doc55. doi: 10.3205/zma001576 (PMC9733484; doi:10.3205/zma001576)
Supplement: Course content and potential additional resources [file JME-39-55-s-001.pdf]

Attachment 1: Course content and potential additional resources

Topic (1) Distributive justice

Introductory article:

“Who Should Get the Last PICU Bed?”[10]

Initial Questions to guide the students’ comments:

1. “Please discuss and debate at least one of the problems in pediatric intensive care presented in the article! Which overarching health care problems are addressed? What options for allocating medical resources are identified? Please comment on these!”
2. “What criteria would you use to triage patients in the event of a shortage of medical resources? Refer to the arguments in the article, but also justify your selection with current examples! In addition to the physician's perspective, try to illuminate the problem from the patient's perspective as well!”

Potential additional resources:

Scientific articles:

“An Ethical Framework for Responding to Drug Shortages in Pediatric Oncology”[25]

“ICU triage in an impending crisis: uncertainty, pre-emption and preparation”[26]

Newspaper articles referring to triage during COVID-19 pandemic

Topic (2) The best interest standard

Introductory article:

“Pepperoni pizza and sex”[11]

Initial Questions to guide the students’ comments:

1. “Please explain at least one of the overarching problems addressed in the article! Comment on the different treatment goals of the parents and the physician! In general, what are the goals of intensive care interventions?”
2. “It is not uncommon for physicians to have different ideas about therapies and treatment goals than patients or their caregivers. Have you encountered such examples yourself? Describe them and discuss how such divergences can best be resolved!”

Potential additional resources:

Scientific articles:

“The Breadth of Hopes”[27]

‘‘We want everything done’’[28]

Video:

Speaking about life and death in the NICU https://www.youtube.com/watch?v=-c06YBQ5jL0

Topic (3) Cross-cultural aspects of pediatric care

Introductory article:

“A Premature Infant With Necrotizing Enterocolitis Whose Parents Are Jehovah’s Witnesses“[12]

Initial Questions to guide the students’ comments:

1. “Please explain at least one of the overarching problems addressed in the article! Take a position on the demands/wishes of the parents! If you had to do the ethical consultation, what would you have advised?”
2. “It is not uncommon for religious and/or cultural factors to play a role in shaping ideas about therapies and therapeutic goals. Have you encountered any such examples yourself? Describe them and address the extent to which medical decisions should depend on them!”

Potential additional resources:

Scientific articles:

“A practical approach to the family that expects a miracle”[29]

“Physicians' Opinions on Engaging Patients' Religious and Spiritual Concerns: A National Survey”[30]

“The Child's Right to an Open Future?”[31]

“Religious aspects of organ transplantation”[32]

Book:

“Kindeswohl“ Ian McEwan

Video:

Interview with Ian McEwan https://www.youtube.com/watch?v=AGuL3JY74A8

Newspaper article:

https://www.newyorker.com/news/news-desk/how-jehovahs-witnesses-are-changing-medicine

Topic (4) Medical futility

Introductory article:

“Trisomy 18 and Complex Congenital Heart Disease: Seeking the Threshold Benefit“[13]

Initial Questions to guide the students’ comments:

1. “Please explain at least one of the overarching problems addressed in the article! Take a position on the demands/desires of the parents! If you had to do an ethical consultation, what would you advise?
2. “When ideas about therapies and therapeutic goals diverge between parents (or patients) and physicians, enormous conflicts can arise. Have you encountered such examples yourself? Describe them and address how (and by whom) these conflicts could be resolved!

Potential additional resources:

Scientific articles:

“Our Children Are Not a Diagnosis: The Experience of Parents Who Continue Their Pregnancy After a Prenatal Diagnosis of Trisomy 13 or 18”[33]

“Parental Hopes, Interventions, and Survival of Neonates With Trisomy 13 and Trisomy 18”[34]

“Perinatal management of trisomy 18: a survey of obstetricians in Australia, New Zealand and the UK”[35]

“Who Is ‘Too Sick to Benefit’?”[36]

Newspaper article:

https://www.tagesspiegel.de/wissen/kinder-mit-trisomie-13-und-18-fuer-tot-erklaert/10661536.html

Video:

https://www.youtube.com/watch?v=kNlr1oyv7mE
