# Supplementary material for: Ethics education in pediatrics: Implementation and evaluation of an interactive online course for medical students
Source: GMS J Med Educ. 2022 Nov 15;39(5):Doc55. doi: 10.3205/zma001576 (PMC9733484; doi:10.3205/zma001576)
Supplement: Summary of the results of the Constructivist On-Line Learning Environment Survey (COLLES) [file JME-39-55-s-002.pdf]

Attachment 2: Summary of the results of the Constructivist On-Line Learning Environment Survey (COLLES). For each scale the average scores on each associated item are presented as mean (blue line) ± SD (vertical bars).

a

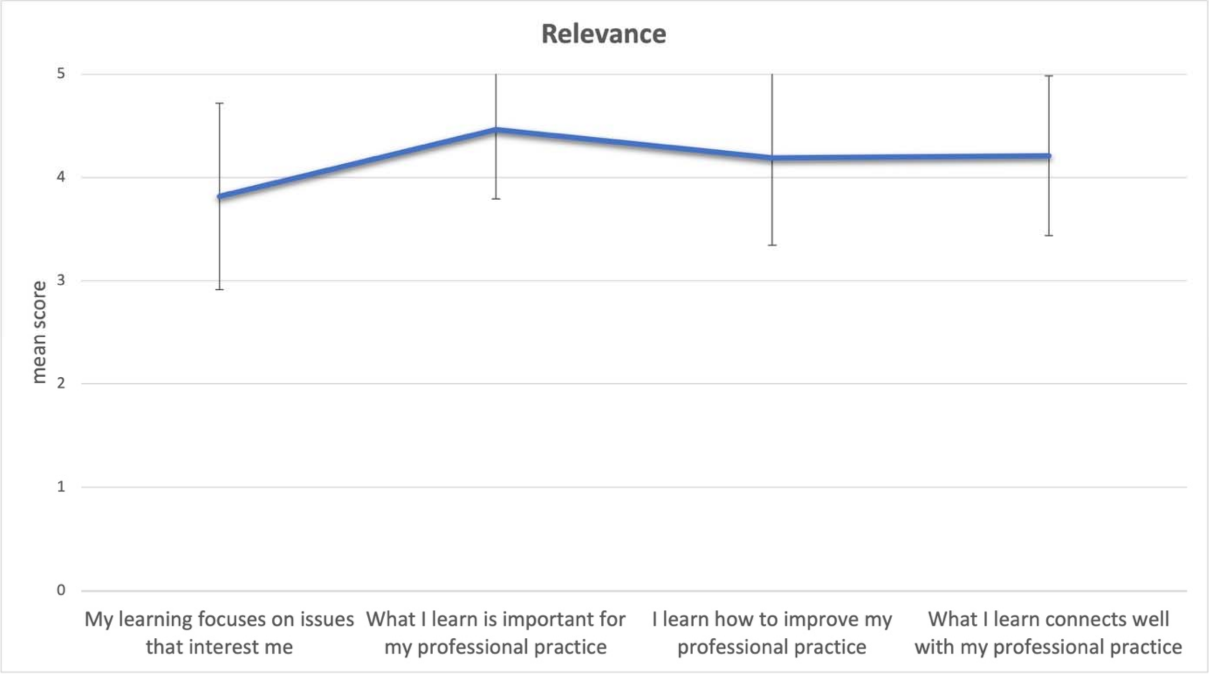

b

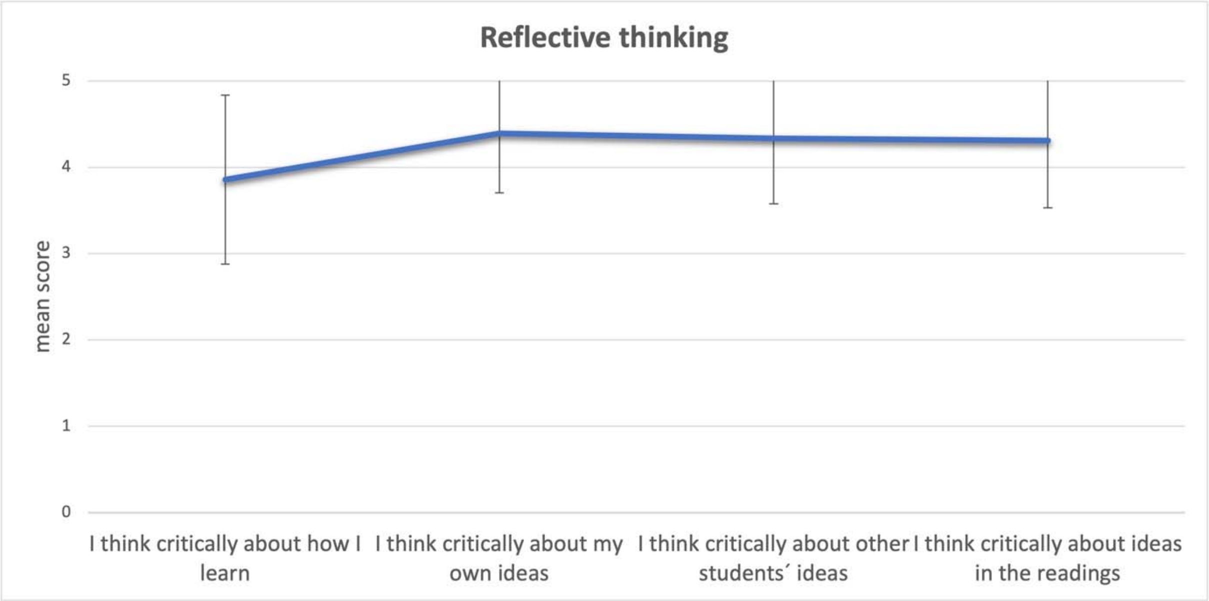

Attachment 2 to Kidszun A, Forth FA, Matheisl D, Busch F, Kaltbeitzel L, Kurz S. *Ethics education in pediatrics: Implementation and evaluation of an interactive online course for medical students*. GMS J Med Educ. 2022;39(5):Doc55. DOI: 10.3205/zma001576

c

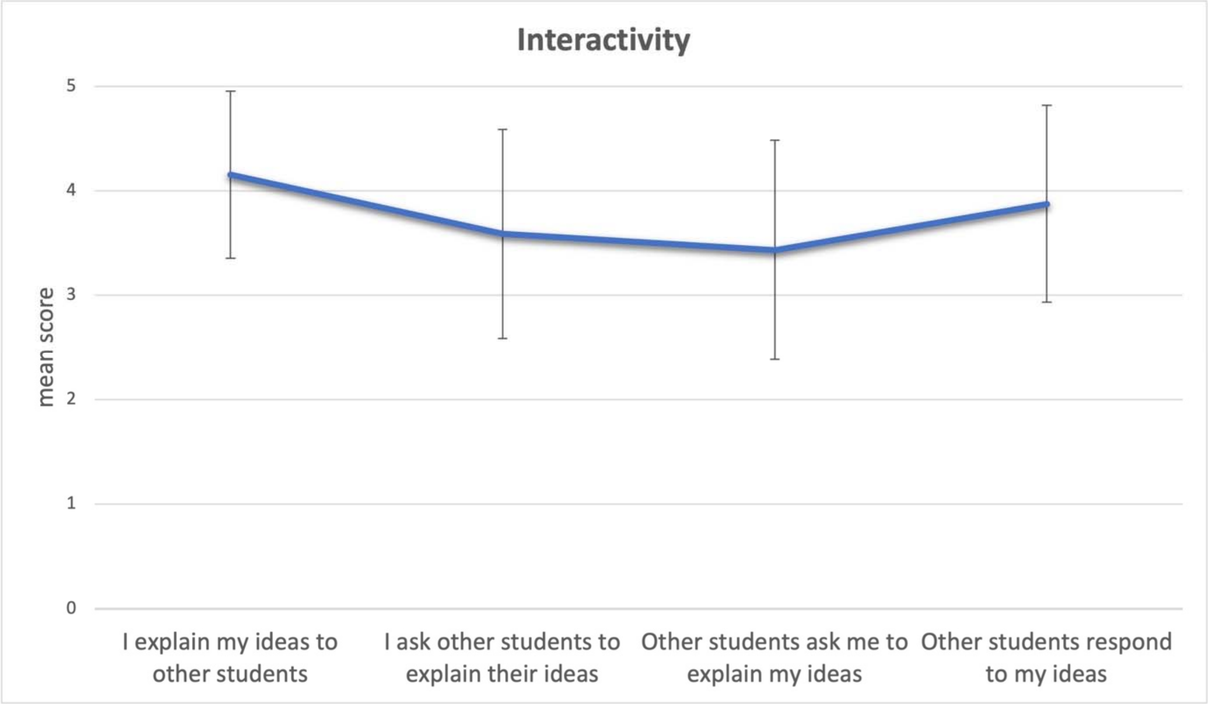

d

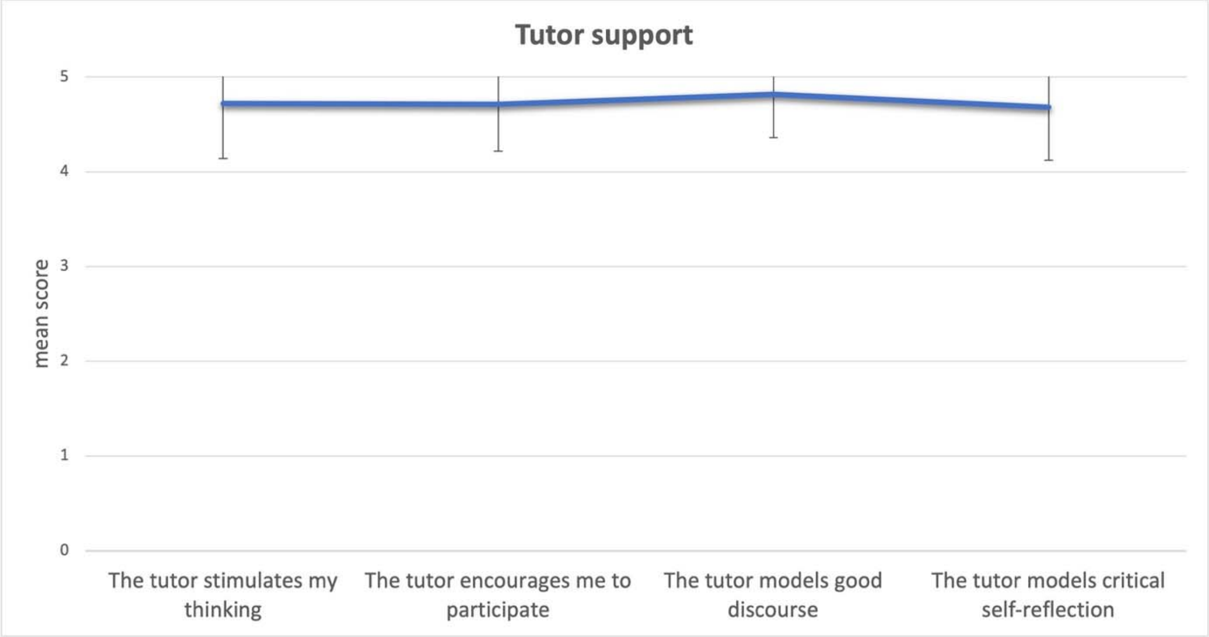

Attachment 2 to Kidszun A, Forth FA, Matheisl D, Busch F, Kaltbeitzel L, Kurz S. *Ethics education in pediatrics: Implementation and evaluation of an interactive online course for medical students*. GMS J Med Educ. 2022;39(5):Doc55. DOI: 10.3205/zma001576

e

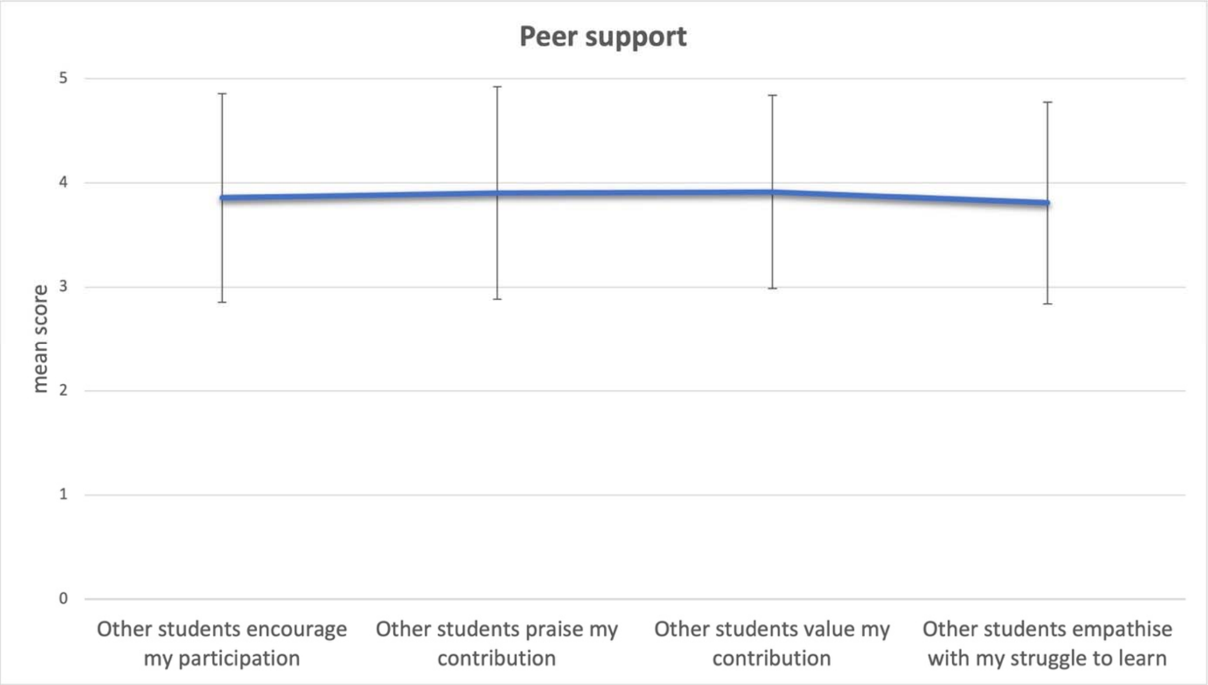

f

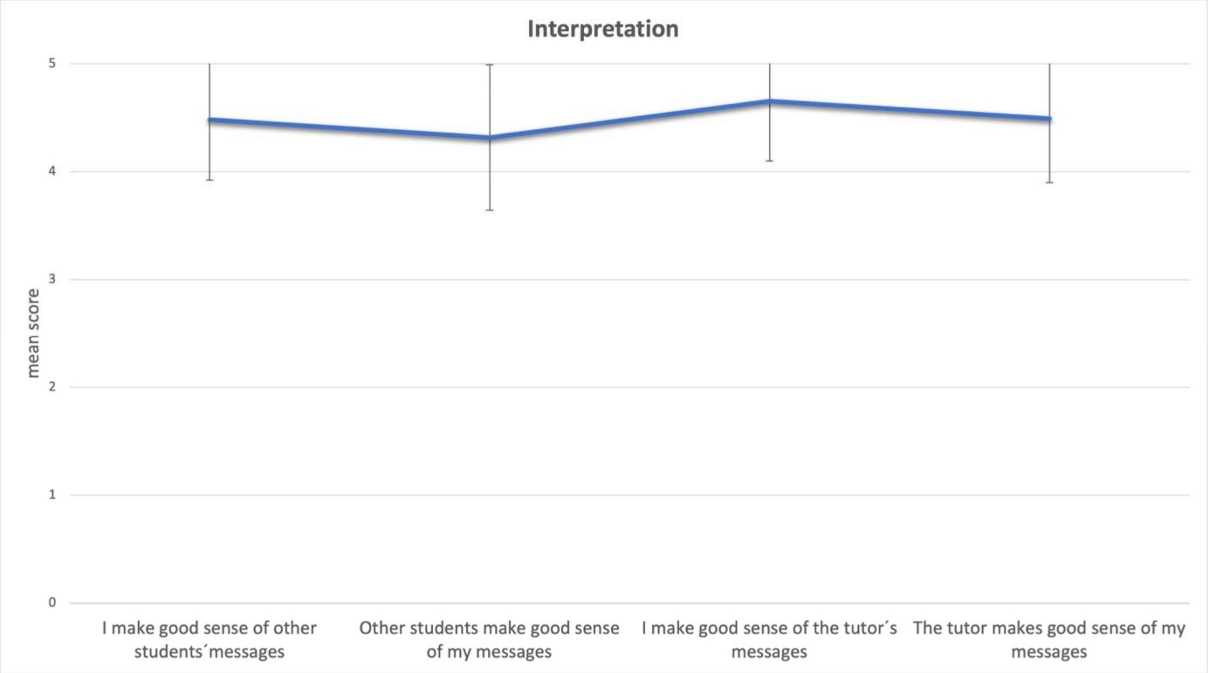

Attachment 2 to Kidszun A, Forth FA, Matheisl D, Busch F, Kaltbeitzel L, Kurz S. *Ethics education in pediatrics: Implementation and evaluation of an interactive online course for medical students*. GMS J Med Educ. 2022;39(5):Doc55. DOI: 10.3205/zma001576

Legend

Diagram 1 a-f summarizes the results of the students' (n=104) self-evaluation for the six domains (scales) of the Constructivist On-Line Learning Environment Survey (COLLES). For the assessment of each of the 24 items a fully verbalized 5-point Likert scale with the following scale points was used: 1 – "almost never", 2 – "seldom", 3 – "sometimes", 4 – "often", and 5 – "almost always". The scores of each scale/domain are presented as mean ± standard deviation (SD). The score range being 1-5, i.e., identical to the number of scale points.

Attachment 2 to Kidszun A, Forth FA, Matheisl D, Busch F, Kaltbeitzel L, Kurz S. *Ethics education in pediatrics: Implementation and evaluation of an interactive online course for medical students*. GMS J Med Educ. 2022;39(5):Doc55. DOI: 10.3205/zma001576
