# Supplementary material for: Ethics education in pediatrics: Implementation and evaluation of an interactive online course for medical students
Source: GMS J Med Educ. 2022 Nov 15;39(5):Doc55. doi: 10.3205/zma001576 (PMC9733484; doi:10.3205/zma001576)
Supplement: Summary of the students’ perceptions derived from the 64 answers on the final open evaluative question: “Do you have any other comments?” [file JME-39-55-s-003.pdf]

Attachment 3: Summary of the students’ perceptions derived from the 64 answers on the final open evaluative question: “Do you have any other comments?”

Students’ perceptions	Number of mentions	Narrative illustration (translated from German)
**Relevance**		
The course is important for my professional practice.	8	“A very creative and innovative approach to learning about a topic that plays as much of a role in a pediatrician's daily clinical practice as classic textbook knowledge.”<br><br>“I think that even though the topic of trisomy 18 was so specific, I still learned something important for my professional future in terms of decision making in a team with patients and relatives.”<br><br>“I found it very interesting to look at these ethical aspects of the practice. Each of us will face similar situations and it is good to look at all points of view and understand the processes behind them.”
Personal development and self-reflection are supported.	5	“The self-reflections and those of others this week will accompany me into my professional life.”<br><br>“The course was very valuable. Nice change from everyday learning and encourages to find one's personal attitude in ethical questions.”<br><br>“Thanks for this opportunity to express ourselves, to take a stand and also to practice self-reflection. This kind of learning should be perceived and performed much more often!”
**Reflective thinking**		
The course stimulates reflective thinking.	19	“[One] is encouraged to think critically about topics, to deal with them and to exchange ideas! So ‘much’ independent thinking and active participation is unfortunately demanded far too rarely in other courses.”<br><br>“It's nice to ‘have to’ think properly for once and not just reproduce memorized knowledge.”<br><br>“The course was very thought-provoking and certainly had a lasting impact on my own way of making decisions.”
A forum is created which allows for personal views, values and attitudes to be formulated and discussed.	5	“It was very interesting to take a closer look at such an important topic and to reflect on it. In a seminar at university, I usually shied away from giving my opinion on such complex and difficult topics. Here I had to think about it, which was good in the end and is also important.”<br><br>“I also appreciated the forum discussion, on the one hand because of the interaction with the other course participants, and on the other hand to further reflect on one's own opinion/attitude.”<br><br>“I particularly liked the open and appreciative discourse and it should definitely continue to be promoted.”

**Active participation**		
Active participation and self-directed learning are encouraged.	9	"This created a wonderful opportunity to exchange ideas and give each other feedback in a very personal way, even online."<br><br>"It inspired me to think extensively and do my own research."<br><br>"I think that this was a good example and to let students take a critical stance for once and not just be passive listeners as is so often the case."
**Role of ethics online education in the medical curriculum**		
The course is well organized. Structure and content work.	15	"I'm surprised how well the forum discussion worked and how well you could exchange ideas online."
Ethics education is important and is not adequately covered in the actual curriculum.	9	"I think the whole idea of the online course was implemented very well and showed us another side of everyday practice that we might not have covered at university, but which is extremely important."<br><br>"The format of the course is really great, because it goes far beyond the mindless reproduction of any learning content and itself stimulates thinking. A process that is very rarely asked for and encouraged in medical school."
The course should be continued in the present format.	6	"Feel free to keep the course the same for future semesters."<br><br>"In my opinion, critical questioning and the intensive discussions were implemented very well. I think this format should also be offered for future semesters."<br><br>"Would like to see exactly this model maintained for everyone after us. I have benefited massively from it."
Constructive alignment is present. The course fits into the curriculum.	3	"I think especially in the final year, where almost all students are about to take their exams and soon go to start practice, such a somewhat difficult topic with partly also fundamental questions of the doctor-patient relationship such as communication, decision making, ethical considerations, etc. should be the focus in many more classes."<br><br>"During our studies, we encounter the topic of 'medical ethics' mainly in the subject 'History, Theory, Ethics of Medicine' in the 6th semester. It is very instructive and refreshing to encounter ethics again in a very applied form. I would continue to incorporate this into the course of study."
This is an innovative and exciting format.	14	"It took until the final year to learn about such an innovative course."<br><br>"Very exciting module. Will stay in my mind."<br><br>"Writing a discussion regarding an English article was new territory for me and I struggled with it a lot in the beginning. However, my mind was changed."

The week-long online format has advantages over classroom and real-time teaching.	4	"I was going to suggest adding a video call to it, but now find that the forum discussion is much more preferable. The biggest advantage with this course was that we the students had enough time to formulate our papers and comments. Therefore, all the papers were very well formulated and focused on the topic. With a face-to-face or video seminar, it would not be possible to have such nice discussions due to the shorter time."<br><br>"Ethical topics have only ever been discussed directly in class at university, which often didn't give you time and space to reflect. In a kind of homework, it gives you even more opportunities to gather."<br><br>"I was given the opportunity to contribute at flexible times."
**Tutor feedback**		
Adequate tutor feedback is important.	7	"I found it encouraging that the tutor provided useful feedback on each post."
**Critics and suggestions for improvement**		
A real-time discussion should be added.	3	"To make it a bit more interactive, an online webinar (on MS Teams) might be a good idea."
This course cannot replace pediatric clinical courses but should be additional.	10	"Of course, it cannot replace the teaching in pediatrics. However, it is a very pleasant change for me to work ethically, discursively for once. In my opinion, this course simply has to be done in addition. In terms of time, this is definitely possible!"<br><br>"I found it very unfortunate that I didn't learn anything subject specific in the course."
More and more different topics should be covered including more practice examples.	7	"The only pity is that we all worked on the same topic, this makes the discussion a bit difficult. If that is possible, it would certainly be interesting to provide different topics for selection"<br><br>"Finally, I would have liked a final statement on how such a situation would have been handled at our local hospital and perhaps one or two examples from here."
